# COVID-19 workplace countermeasures that occupational physicians could not change in Japan: a qualitative study

**DOI:** 10.1186/s12889-024-21219-9

**Published:** 2025-01-08

**Authors:** Yu Igarashi, Seiichiro Tateishi, Juri Matsuoka, Tomoko Sawajima, Mika Kawasumi, Arisa Harada, Koji Mori

**Affiliations:** 1 Disaster Occupational Health Center, Institute of Industrial Ecological Sciences, University of Occupational and Environmental Health, 1-1 Iseigaoka, Yahatanishi-ku, Kitakyushu, Fukuoka 807-8555 Japan; 2 HOYA CORPORATION, Tokyo, Japan; 3 Health Care Center, Tokyo Health Care Office, Central Japan Railway Company, Tokyo, Japan; 4 Department of Occupational Medicine, School of Medicine, University of Occupational and Environmental Health, Fukuoka, Japan; 5 Department of Occupational Health Practice and Management, Institute of Industrial Ecological Sciences, University of Occupational and Environmental Health, Fukuoka, Japan

**Keywords:** COVID-19, Japan, Workplace countermeasures, Occupational health, Occupational physician, Workers, Workplace

## Abstract

**Background:**

During the COVID-19 pandemic, information and circumstances changed from moment to moment, including the accumulation of scientific knowledge, the emergence of variants, social tolerance, and government policy. Therefore, it was important to adapt workplace countermeasures punctually and flexibly based on scientific evidence and according to circumstances. However, there has been no assessment of changes in workplace countermeasures. With a view toward preparedness for future pandemics, we surveyed COVID-19 workplace countermeasures that occupational physicians considered as needing to be changed but went unchanged.

**Methods:**

We invited 685 professional occupational physicians certified by Japan Society for Occupational Health to complete an online questionnaire by sending postcards with QR codes. The main questions concerned countermeasures that the participants wanted to change but could not. The survey period was from February 21 to April 28, 2022. The responses were analyzed using the KJ method.

**Results:**

Of the 168 invitees (24.5%) who responded to the survey, 125 reported countermeasures that needed to be changed (total count: 254). The responses were categorized into basic systems, occupational health services, workplace countermeasures, vaccines, and incidents, with a code count of 7, 8,147, 10, and 82, respectively. The type of countermeasure was 115 for countermeasures to be strengthened (CBS), 110 for measures to be mitigated (CBM), and 29 for neither.

**Conclusions:**

Often-mentioned CBS were increased teleworking, strengthened ventilation, smoking cessation, and promotion of vaccines. Often-mentioned CBM were relaxation of protective equipment rules, discontinued environmental disinfection, and shorted isolation and reinstatement. In the early pandemic phases, CBSs were frequently mentioned, whereas CBMs were featured more prominently in the latter phases. The survey revealed countermeasures that occupational physicians thought needed to be changed but were not changed in practice. For future responses to emerging and reemerging infectious diseases, it will be necessary to establish rules compatible with flexible modification of workplace countermeasures in response to changing circumstances.

## Background

The pandemic of the Coronavirus disease 2019 (COVID-19) has had an enormous impact worldwide [1], and countermeasures to prevent infection at workplaces, which have become one of the places where the infection has spread, have become important [2–4]. Workplace countermeasures include wearing masks, ventilation, hand washing, teleworking, online meetings, home isolation, antigen testing, and vaccination [5–7].

Workplace countermeasures may interfere with the performance of work or create new health issue for workers. These health issues include loneliness [6] and lack of telework support [7], mental health problems from overwork [8], skin conditions [9] and communication errors [10] due to mask-wearing, and lifestyle diseases from reduced exercise during isolation [11]. For this reason, a balanced approach is essential, considering both the risk of infection-related health impacts and the potential negative effects of countermeasures, while avoiding excessive measures.

According to ISO 31,000, risk is defined as the effect of uncertainty [12].　 Uncertainty around emerging infectious diseases often stems from an initial lack of information about a pathogen at the onset of an outbreak [13]. Consequently, risks are generally assessed as high in the early stages, subsequently declining as greater knowledge about the pathogen reduces uncertainty [14]. In the case of COVID-19, infection-related health risks gradually decreased due to the spread of low-pathogenic variants [15], the widespread adoption of PCR and antigen tests (16–17), and advancements in vaccines [18] and treatments [19]. Thus, timely adjustments to workplace COVID-19 countermeasures, informed by evidence and current circumstances, were essential. Nonetheless, in practice, various factors may resist flexible adaptation to changing risks and requirements. To our knowledge, no studies have examined the specific countermeasures necessary to change and the extent of their practical implementation.

In Japan, companies with over 50 employees are legally required to appoint occupational physicians (OPs) to lead health initiatives [20]. OPs are uniquely positioned to have the most comprehensive knowledge of both infectious diseases and workplace conditions. It has been reported that they played numerous roles in implementing countermeasures against COVID-19 [21]. Consequently, OPs were expected to gather and interpret scientific evidence, apply it in alignment with organizational needs and operational realities, and propose flexible adjustments to countermeasures for employers as necessary [22].

Therefore, in this study, we investigated the countermeasures that could not be modified, based on the assumption that the adjustments OPs believe should be implemented reflect actual needs. We believe that the results of this survey provide important insights for the implementation of effective countermeasures in future pandemics.

## Methods

### Participants selection and survey procedures

The subjects of this study were OPs. In Japan, most OPs have received only minimal training, but some have undergone systematic training in occupational health and passed the certification exam administered by Japan Society for Occupational Health (JSOH) to become JSOH-certified professional OPs, most of whom work full-time in occupational health [20]. In this study, the evaluation of changing needs is based on the perspectives of OPs. To enhance the validity of assessing changing needs in the workplace, the study participants were limited to JSOH-certified professional OPs. We obtained mailing labels for 689 Specialist OPs from JSOH and sent postcards with QR codes. A total of 689 postcards were sent. We did not issue a mass reminder due to difficulties in reacquiring mailing labels and the anonymity of the participants. Instead, we sent a reminder email using the mailing list to which the majority of JSOH-certified professional OPs are registered. The questionnaire used a Google form, and responses were anonymous. The discontinuation criterion was a statement of decline to participate. The survey ran from February 21 to April 28, 2022.

### Survey contents

The survey items included attributes such as age, experience as a doctor and as an OP, open-ended questions about countermeasures that should be changed but could not be changed (CBC: countermeasure to be changed), type of change wanted (strengthen, mitigation, neither), when change was attempted. The time period is divided uniquely according to the number of infected cases in Japan: (1) first wave and its convergence period (January to June 2020), (2) second wave and its convergence period (July – October 2020), (3) third wave and its convergence period (November 2020 – February 2021), (4) fourth wave and its convergence period (March 2021-June 2021), (5) fifth wave and its convergence period (July 2021-October 2021), and (6) sixth wave (November 2021- the time of reply). The CBC-related question was: “As an OP, what countermeasures did you want to change but could not during the COVID-19 pandemic?” The question was originally developed for this study.

### Analysis

The answers to CBC questions were coded, leaving their essential meanings intact. Those with unclear content, or unrelated to workplace countermeasures, were excluded. Codes containing multiple meanings were split into two. For qualitative content analysis, codes were categorized by the KJ method and Berelson’s concept [23]. A category is a group of words with similar meanings. First-, second-, and third-level categories were broken down from abstract to concrete. The KJ method was performed by the first group of researchers (KM, JM, YI), then the second (ST, AH), with inter-rater checks performed for all categories. Discrepancies were resolved by the first group of researchers accepting the second group’s category decisions or by discussing and resolving disagreements. Concerning types of CBC, a “countermeasure to be strengthened (CBS)” was defined as creating rules, restricting some actions, and increasing the number of procedures to be implemented. For example, it could include establishing internal guidelines, promoting teleworking, wearing masks, changing hand sanitizers, closing smoking areas, recommending vaccinations, and restricting attendance of anyone suspected of being infected. A “countermeasure to be mitigated (CBM)” was defined as changing by reducing, shortening, suspending, or abolishing currently active countermeasures. For example, it could include easing restrictions on coming to the office or on business trips, shortening the period of isolation at home, discontinuing antigen testing for those entering the premises, discontinuing environmental disinfection, or abolishing the prohibition on tooth brushing. The “Neither” category included countermeasures that were unclear regarding whether they were strengthening or mitigating types or whether they included both aspects, and implementation or discontinuation of countermeasures for which there was insufficient scientific evidence. Examples of this category include flexible modification of countermeasures, role-sharing, and discontinuation of purchasing space sterilization equipment. Antigen testing and vaccine testing packages for asymptomatic persons were excluded, because the participants’ intent to strengthen or mitigate was not clear. In the event that participants’ selections (strengthen, mitigation, neither) differed from the researchers’ definitions, types were modified according to the latter definitions.

### Ethical consideration

All procedures in this study were performed in accordance with the relevant guidelines and regulations in the Declaration of Helsinki. The study was approved by the Ethics Committee of the University of Occupational and Environmental Health, Japan (Approval numbers: R2-020). Informed consent was obtained in the form of completed questionnaires from all participants.

## Results

Responses were received from 165 participants (24.1% response rate). Table 1 shows the characteristics of participants. The participant’s demographic was 71.5% men, with a mean age of 47.7 years (SD = 10.6), and 35.8% in their 40s. The mean years of experience as a doctor was 22.3 years (SD = 10.3), with 21.2% having 16–20 years of experience, while the mean years of experience as an OP was 17.6 years (SD = 9.1), with 18.8% reporting 16–20 years of experience. Forty participants reported no countermeasures to be changed, 28 indicated one countermeasure, 52 noted two countermeasures, and 45 identified three or more countermeasures.

**Table 1:** Characteristics of participants

	Total (*n*=165)	%
Age, n (%)		
-39	43	26.0
40-49	59	35.8
50-59	34	20.6
60-	29	17.6
Sex, n (%)		
Men	118	71.5
Women	47	28.5
Years of expericence physician, n (%)		
-5	11	6.7
6-10	32	19.4
11-15	31	18.8
16-20	35	21.2
21-25	24	14.5
26-	32	19.4
Years of expericence as an Occupational physician, n (%)		
-10	24	14.5
11-15	24	14.5
16-20	31	18.8
21-25	26	15.8
26-30	26	15.8
31-	34	20.6
Coutermeasures to be changed, n (%)		
0	40	24.2
1	28	17.0
2	52	31.5
3 or more	45	27.3

Table 2 showed category, second category, third category, types of changes, example of codes and each number of codes The first-level category was divided into basic systems, occupational health services, workplace countermeasures, vaccines, and incidents.

The first-level category “Basic system,” included “Rule,” “Flexible change” and “Role” as the second-level category. There was no third-level category. Frequently mentioned items in the second-level category were “Rules” for CBS (three codes) and “Flexible changes” for the “Neither” type (two codes).

**Table 2:** Category, second category, third category, types of changes, example of codes and each number of codes

First category	Second category	Third category	Type of changes	Examples of codes that OPs wanted to change but could not	Number of codes
Basic system	Rule Flexible change Role		Strengthen	Establishment of internal countermeasures guidelines	3
	Flexible change		Neither	Flexible modification of countermeasures in response to pandemic conditions	2
	Role		Neither	Revising the vertical division of roles for countermeasures	2
Occupational health service	Online interview		Strengthen	(Occupational health staff) online work (promotion)	4
	Service delivery		Strengthen	Visits to workplaces (on-site guidance for countermeasures)	4
Workplace countermeasures	Questionable countermeasures	Space sterilization	Neither	Discontinuation of purchase of space sterilization equipment	9
	Testing	Antigen test・PCR	Strengthen	Implementation of PCR testing	3
			Mitigation	Discontinuation of antigen testing (for all workers) upon entry to workplaces	8
			Neither	Antigen testing of asymptomatic workers (unknown intent)	4
	Rules of conduct	Partition	Mitigation	Removal of partitions	4
		Tooth brushing	Mitigation	Abolition of the rule prohibiting tooth brushing	2
		Hand disinfection	Strengthen	Change from hypochlorite water to alcohol	8
			Mitigation	Abolition of rule prohibiting the use of jet towels	4
		Body temperature measurement	Mitigation	Abolition of the rule for non-contact body temperature measurement upon entry (of employees and visitors) to the building	1
		PPE	Strengthen	Designation of (type of) non-woven masks	18
			Mitigation	Removal of masks depending on the situation (Abolition of the rule of always wearing a mask)	5
	Rules restricting work and travel	Rule on moving	Strengthen	Restrictions on business travel	3
			Mitigation	Removal of travel restrictions to local factories	5
			Neither	Vaccine testing package (unknown intent)	2
		Rule on eating outside	Strengthen	Restrictions on eating, drinking outside, and karaoke	2
			Mitigation	Mitigation of rules limiting the number of dinners	2
		Teleworking	Strengthen	Promotion of teleworking	13
		Rule on meeting	Strengthen	Implementation of online meetings	2
	Rules in the work environment	Ventilation	Strengthen	Installation of CO2 monitors to assess (and improve) ventilation conditions	11
		Smoking	Strengthen	smoking cessation for smokers	3
			Mitigation	Abolition of no-smoking rule in the workplace	2
		Rule on using facilities	Strengthen	Closure of smoking areas	21
			Mitigation	Abolition of outdoor activity restrictions	1
			Neither	Timing of changes to restrictions on use of in-house facilities	1
		Environmental disinfection	Strengthen	Change of linen for napping beds	1
			Mitigation	Discontinuation of environmental disinfection of common areas	9
	Individualized support	High risk person	Strengthen	Promotion of teleworking for those with serious illnesses	2
		Childcare employees	Neither	Promotion of work-related support for childcare employee	1
Vaccine			Strengthen	Promotion of vaccination	8
			Mitigation	Discontinuation of strong recommendations for vaccination	2
Incident	Whole group	Isolation and reinstatement	Mitigation	Shortening of the period of restricted working hours	8
	Infected persons	Isolation and reinstatement	Mitigation	Discontinuation of the 2-week activity record rule	23
			Neither	Flexible operation of the stay-at-home period	6
	Persons in close contact	Isolation and reinstatement	Strengthen	Establishment of virus testing rules	1
			Mitigation	Shortening of the waiting period for persons in close contacts	18
			Neither	Flexible operation of the waiting period at home	2
		Definition	Strengthen	Establishment of criteria for identification of person in close contacts	1
			Mitigation	Elimination of identification of person in close contacts	7
	Persons in close contact with person in close contact	Isolation and reinstatement	Mitigation	Elimination of home-waiting instructions for contacts of person in close contacts	5
	Person with symptom	Isolation and reinstatement	Strengthen	Establishment of home isolation rules for PCR-negative symptomatic persons	7
			Mitigation	Shortening the isolation period for vaccinated persons	4
				Total	254

PCR: polymerase chain reaction, PPE: personal protective equipment, OP: Occupational Physician

The first-level category “Occupational health services,” included “Online Interviewing” and “Service delivery” as second-level categories. There was no third-level category. Frequently mentioned items in the second-level category were “Online interviews” for CBS (four codes) and “Service delivery” for CBS (four codes).

The first-level category “Workplace countermeasures,” included “Questionable countermeasures,” “Testing,” “Rules of conduct,” “Rules restricting work and travel,” “Rules in the work environment,” and “Individualized support” as the second-level category. Frequently mentioned items in the third-level category were “Space disinfection” for the “Neither” type (second-level category: Questionable countermeasures, nine codes), “PPE (personal protective equipment)” for CBS (second-level category: Rules of conduct, 18 codes), “Teleworking” for CBS (second-level category: Rules restricting work and travel, 13 codes), “Ventilation” for CBS (second-level category: Rule in the work environment, 11 codes), “Rule on using facilities” for CBS (second-level category: Rule in the work environment, 21 codes), and “Environmental disinfection” for CBM (second-level category: Rule in the work environment, eight codes).

For the first level category “Vaccine,” there were no second or third-level categories, and the most frequently mentioned item was “Vaccine” in the CBS (eight codes).

The first-level category “Incidents,” included “Whole group,” “Infected persons,” “Persons in close contact,” “Persons in close contact with persons in close contact,” and “Symptomatic persons” as the second-level category. Frequently mentioned items in the third-level category were “Isolation and reinstatement” for CBM (second-level category: Infected persons, 23 codes), “Isolation and reinstatement” for CBM (second-level category: Persons in close contact, 18 codes), and “Isolation and reinstatement” for CBM (second-level category: Whole group, eight codes).

### Results by phase

**FIgure. 1:**
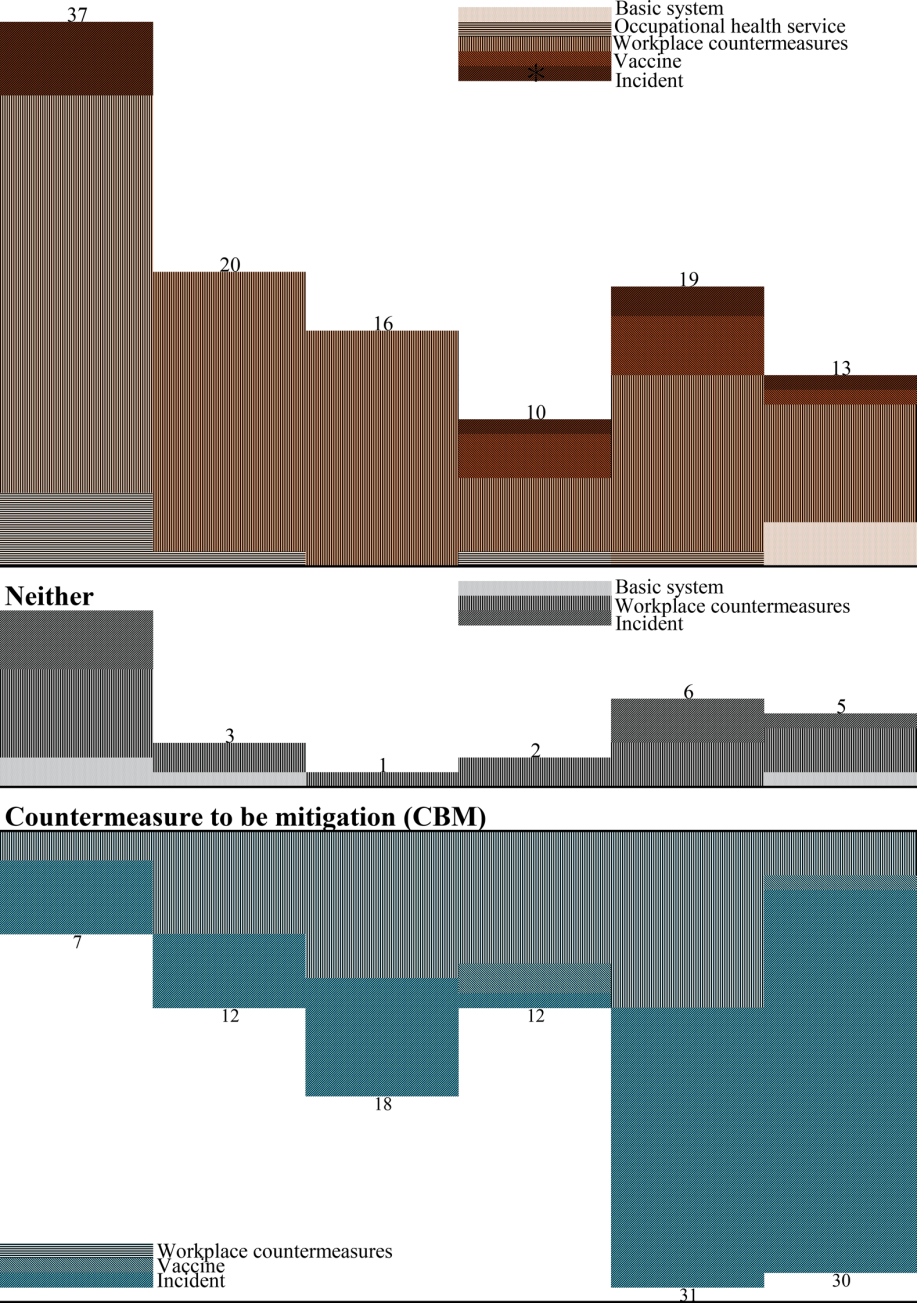
Countermeasures to be changed, the type of change, and the phase of each. First wave Second wave Third wave Forth wave Fifth wave Sixth wave Countermeasure to be strengthened (CBS)

Table 3; Fig. 1 shows categories of change countermeasures to be changed, the type of change, and the phase of each; these were: 56 for the first wave (37 for CBS, 7 for CBM, 12 for Neither), 35 for the second wave (20, 12, 3), 35 for the third wave (16, 18, 1), 24 for the fourth wave (10, 12, 2), 56 for the fifth wave (19, 31 6), and 48 for the sixth wave (13, 30, 5). A trend emerged, indicating a greater emphasis on CBS in the early phases, shifting towards more CBM in later phases.

**Table 3:** Categories of change countermeasures to be changed, the type of change, and the phase of each

First category	Type of change	First wave	Second wave	Third wave	Forth wave	Fifth wave	Sixth wave	Total
Basic system	Strengthen						3	3
	Neither	2	1				1	4
Occupational health service	Strengthen	5	1		1	1		8
Workplace countermeasures	Strengthen	27	19	16	5	12	8	87
	Mitigation	2	7	10	9	12	3	43
	Neither	6	2	1	2	3	3	17
Vaccine	Strengthen				3	4	1	8
	Mitigation				2			2
Incident	Strengthen	5			1	2	1	9
	Mitigation	5	5	8	1	19	27	65
	Neither	4				3	1	8
Total (type)	Strengthen	37	20	16	10	19	13	**115**
	Mitigation	7	12	18	12	31	30	**110**
	Neither	12	3	1	2	6	5	**29**
**Total**		**56**	**35**	**35**	**24**	**56**	**48**	**254**

## Discussion

This study used a qualitative analysis of an online questionnaire survey to identify workplace countermeasures that OPs believed should be changed during the COVID-19 pandemic but that went unchanged. Often-mentioned “to be strengthened” items (CBS) were increased teleworking, strengthened ventilation, smoking cessation, and promotion of vaccines. Often-mentioned “to be mitigated” items (CBM) were relaxation of protective equipment rules, discontinued environmental disinfection, and shorted isolation and reinstatement. In the early pandemic phases, CBSs were frequently mentioned, whereas CBMs were featured more prominently in the latter phases. This is the first study to investigate failures to flexibly adapt workplace countermeasures in the COVID-19 pandemic; the findings concerning insufficient and excessive countermeasures may be of value for future pandemics.

Although many studies identified effective workplace countermeasures during the COVID-19 pandemic [4, 5, 24], gaps remained in their implementation. OPs initially emphasized strengthening countermeasures but later shifted to mitigating them as risks became clearer [15]. OPs advised employers or the person in charge of countermeasures to strengthen safety measures early on and adjust them as uncertainty decreased. However, as this study shows, it may not be easy to realize such flexible changes in the workplace: there were many countermeasures that OPs tried to change but could not. In other words, the advice from the OP’s medical risk perspective was not always adopted as is in infection control decision-making; companies are considered to have assessed the overall risk, including management and reputational factors.

Reasons for the occurrence of the “desired-realized” gap for CBS might include company hesitancy about introducing strict measures due to costs, and because of concerns about employee rights, lower performance due to implementing new, strict rules in an uncertain situation, and the inability to apply the rules to everyone (e.g., due to concerns about employees with health conditions). By contrast, the reason for the gap for CBM might be difficulty in deciding to mitigate countermeasures now in place; in particular, large companies involved in social infrastructure are vulnerable to social criticism. In some companies, countermeasures are sometimes based upon unreliable information [25], but the practices may not be easily changed, for example, simply at the discretion of the company or the level of the person in charge. Another possible reason could be that the OP’s opinion was not considered important to the policymakers. This is not only in terms of risk assessment, but also in terms of the possibility that the relationship between OPs and decision makers before COVID-19 pandemic may have been weak or not included in the organization’s decision-making structure. Professionals must be aware of the various difficulties regarding workplace countermeasures in an emerging infectious disease pandemic.

Inappropriate countermeasures might be a source of significant problems. Insufficient measures may lead to infection clusters in the workplace because the risk of infection is not adequately addressed. However, excessive countermeasures can cause other adverse effects, including, for example, costs in time and effort due to excessive environmental disinfection, communication errors [11], skin disorders [10] due to mask-wearing, mental health problems [9], and reduced labor productivity [26] due to long home isolation. It is necessary to avoid implementing insufficient, incorrect, or excessive countermeasures; instead, countermeasures should be reviewed and changed flexibly based on correct information to minimize negative impacts on business and employee health.

Among the results of the survey, the two items in the first-level category with the highest number of codes: “Countermeasure at the workplace” (147 codes, 57.9%) and “Incidents” (82 codes, 32.2%), are discussed in detail below, with reference to the background to the desired change and reasons for the difficulties in implementing change.

### Workplace countermeasures

Scientific evidence regarding masks, ventilation, disinfectants, and antigen tests [18, 27–29] highlighted the need for countermeasure adjustments, yet gaps between evidence and practice persisted. Initially, masks were primarily worn by symptomatic individuals, but as droplet and aerosol transmission became evident [30], widespread mask-wearing was strongly encouraged [31]. Despite evidence favoring non-woven masks [32], enforcing rules on mask types proved challenging, as some employees resisted due to skin issues or cosmetic reasons. Improvements to ventilation, though scientifically supported [28], were hindered by costs, comfort concerns, and pollen prevention. Hypochlorous acid water, initially used due to an alcohol shortage, was later deemed ineffective without sufficient concentration [33], yet some companies continued its use, ignoring OP recommendations to switch to alcohol-based disinfectants. Space sterilization, despite official warnings [34], also persisted in some workplaces. Antigen and PCR tests were used to prevent workplace infections and provide negative proof for travel and business trips. Initially questioned, antigen testing was later shown to be effective [18] and widely adopted by companies. Particularly for business trips and large construction projects, some OPs aimed to utilize tests effectively, while others sought to prevent misuse and address the issue of excessive testing of asymptomatic individuals, which led to a flood of unnecessary negative certificates.

### Incidents

The emergence of variants and increasing knowledge about COVID-19 necessitated repeated updates to isolation periods for infected individuals. The Japanese government revised isolation periods from 14 to 10 days (June 2020), 7 days (September 2022), and 5 days (May 2023). Isolation for “persons in close contact” was similarly shortened based on medical findings, social tolerance, and workforce shortages. In Japan, the definition of “persons in close contact” was revised in July 2021 (5th wave) from those in contact with an infected person from the day of symptom onset within 2 m without precautions (e.g., masks) to those in contact from 2 days before symptom onset within 1 m without precautions (35–36). Initially, public health authorities managed these measures [36], but as case numbers surged (e.g., January 2021), companies had to create their own definitions and policies, often exceeding government guidelines in safety. In this study, codes for “infected persons” and “persons in close contact” per wave were 5-0-3-0-9-12 and 2-1-3-0-9-14, respectively. Early phases focused on requiring PCR testing (Neither), while later phases highlighted excessive isolation periods (CBM), which often remained unaddressed despite OPs’ concerns.

When preparing infection control measures for the next pandemic, it is crucial to establish a system to adjust countermeasures appropriately based on updated information. This includes defining the roles and authorities of OPs and other specialists, creating mechanisms for stakeholder meetings, and ensuring communication with decision-makers. The COVID-19 pandemic highlighted the need to adapt workplace countermeasures to various changes, including pathogen characteristics, countermeasure effectiveness, tests, vaccines, treatments, social tolerance, and government policies. The “Hammer and Dance” theory advocates strict measures like lockdowns during high infection periods, while mitigating restrictions and resuming near-normal activities during low infection periods [37]. Infection control should adjust repeatedly to infection waves. This study revealed workplace countermeasures often lacked flexibility. Provisions for flexible modification of countermeasures should be included in BCPs (business continuity plan) and manuals prepared for the next pandemic. Specifically, it is essential to establish action guidelines that regularly review the procedures for obtaining information, evaluating and deciding on countermeasures, and implementing them. These guidelines should be shared among all stakeholders, including the government, companies, and professionals.

### Limitations

This study has several limitations. One is recall bias: we asked about past responses to COVID-19. However, we consider this negligible, as the survey was conducted during the pandemic. Another issue is selection bias: as we surveyed occupational health specialists certified by the Japan Society for Occupational Health, the study population does not reflect all OPs in Japan. However, the fact that we discovered many issues that even certified specialists could not change is consistent with the purpose of our survey. Third, the survey response rate was relatively low (24.5%) representing only 168 participants. It is possible that those who did not respond did not have any countermeasures that they could not change. Nonetheless, our survey has revealed the phenomenon of OPs wanting to change workplace countermeasures but being unable to accomplish their goals. It is also possible that the present findings were collected from relatively large companies, or companies with a high level of health and safety activities, such as companies that appoint JSOH-certified professional OPs. Small and medium-sized companies reportedly implemented fewer countermeasures than large companies [38], and it is conceivable that smaller companies instigated more inefficient countermeasures. Finally, there are limitations to generalizing our findings due to the focus solely on Japanese companies and participants. However, the workplace countermeasures considered here appear universal [4, 5, 24], along with some of the obstacles to changing countermeasures, such as resistance to wearing masks or vaccination [39, 40] as well as cost issues. Although the end of the public health emergency of international concern (PHEIC) was declared by WHO in May 2023, the epidemic of infection is still in progress, and it is unclear what will happen to settle it, and countermeasures remain a big issue. Further research is needed to better identify appropriate and flexible changes in workplace countermeasures, compatible with the different pandemic-related scenarios.

## Conclusions

We have identified difficulties in flexibly changing workplace countermeasures by examining countermeasures that OPs wanted to change but were unable to change during the COVID-19 pandemic. Our findings may help to improve workplace countermeasures in the next pandemic.

## Data Availability

The datasets generated and analyzed during the current study are available from the corresponding author on reasonable request.

## Abbreviations

WHO: World Health Organization
PCR: Polymerase chain reaction
OP: Occupational physician
JSOH: Japan Society for Occupational Health
CBS: Countermeasure to be strengthened
CBS: Countermeasure to be mitigated
PHEIC: Public health emergency of international concern
BCP: Business continuity plan
